# Methyl Benzoate Is Superior to Other Natural Fumigants for Controlling the Indian Meal Moth (*Plodia interpunctella*)

**DOI:** 10.3390/insects12010023

**Published:** 2020-12-31

**Authors:** Md Munir Mostafiz, Errol Hassan, Rajendra Acharya, Jae-Kyoung Shim, Kyeong-Yeoll Lee

**Affiliations:** 1 Division of Applied Biosciences, College of Agriculture and Life Sciences, Kyungpook National University, Daegu 41566, Korea; munirmostafiz12@gmail.com (M.M.M.); racharya2048@gmail.com (R.A.); astelia@naver.com (J.-K.S.); 2 School of Agriculture and Food Sciences, The University of Queensland Gatton, Queensland 4343, Australia; e.hassan@uq.edu.au; 3 Institute of Agricultural Science and Technology, Kyungpook National University, Daegu 41566, Korea; 4 Institute of Quantum Dot Fusion Science and Technology, Kyungpook National University, Gunwi 39061, Korea

**Keywords:** stored-product insect, fumigation toxicity, naturally available compound, monoterpenes

## Abstract

**Simple Summary:**

Globally, the Indian meal moth is an insect pest of stored goods and manufactured foodstuffs. Synthetic fumigants, such as phosphine and methyl bromide, are widely used agents to control this species. However, due to the development of resistance and increasing concern about the potential adverse effects of synthetic fumigants, it is now necessary to identify environmentally friendly alternatives. Naturally occurring compounds, such as essential oils (EOs), are perhaps the most promising alternative sources; many have been successfully used as active ingredients in contact-based control products, repellents, and fumigants. Methyl benzoate (MBe) is an environmentally friendly, food-safe, natural insecticide that offers a possible alternative to synthetic equivalents. Here, we evaluated the fumigant toxicity of MBe against adults of the Indian meal moth and found that it had great potential for the control of these insect pests in stored products.

**Abstract:**

The Indian meal moth, *Plodia interpunctella* (Hübner) (Lepidoptera: Pyralidae), is an insect pest that commonly affects stored and postharvest agricultural products. For the control of insect pests and mites, methyl benzoate (MBe) is lethal as a fumigant and also causes contact toxicity; although it has already been established as a food-safe natural product, the fumigation toxicity of MBe has yet to be demonstrated in *P. interpunctella*. Herein, we evaluated MBe as a potential fumigant for controlling adults of *P. interpunctella* in two bioassays. Compared to the monoterpenes examined under laboratory conditions, MBe demonstrated high fumigant activity using a 1-L glass bottle at 1 μL/L air within 4 h of exposure. The median lethal concentration (LC_50_) of MBe was 0.1 μL/L air; the median lethal time (LT_50_) of MBe at 0.1, 0.3, 0.5, and 1 μL/L air was 3.8, 3.3, 2.8, and 2.0 h, respectively. Compared with commercially available monoterpene compounds used in pest control, MBe showed the highest fumigant toxicity (toxicity order as follows): MBe > citronellal > linalool > 1,8 cineole > limonene. Moreover, in a larger space assay, MBe caused 100% mortality of *P. interpunctella* at 0.01 μL/cm^3^ of air after 24 h of exposure. Therefore, MBe can be recommended for use in food security programs as an ecofriendly alternative fumigant. Specifically, it provides another management tool for curtailing the loss of stored food commodities due to *P. interpunctella* infestation.

## 1. Introduction

The Indian meal moth, *Plodia interpunctella* (Hübner) (Lepidoptera: Pyralidae), is a cosmopolitan pest that infests a wide range of stored and postharvest products including cereal products, dried fruits, nuts, and legumes [1,2]. Historically, this species is among the world’s major economically important insect pests of raw and manufactured agricultural products, warehouses, and retail environments [3,4,5]. For example, its larvae are associated with 179 different food commodities in 48 different countries across six continents [6]. Infestations of *P. interpunctella* can cause direct commodity loss as well as indirect economic costs through pest control, quality losses, and consumer complaints [7]. Given that insect allergens now represent a serious threat to human health, contamination of commodities by *P. interpunctella* is also an important issue for modern allergy-sensitive communities [8]. Over the past few decades, control of *P. interpunctella* has relied heavily on the use of synthetic pesticides (e.g., organophosphates and pyrethroids) and fumigants (e.g., methyl bromide or phosphine) [9,10].

Fumigants have typically been used to control stored-product insect pests in large commodities, packaged materials, and structures; hence, methyl bromide and phosphine are widely used as fumigation control agents. Both compounds are highly effective for pest control; however, their high mammalian toxicity makes them hazardous to work with during large-scale fumigations, especially in confined spaces [11]. Furthermore, methyl bromide has been declared an ozone-depleting substance, and its use is currently being phased out [11,12]. Phosphine is currently used to control adults of stored-product pests, which are mainly controlled in open spaces such as in flour mills [13]; however, insect resistance to phosphine is now a global issue, with control failures having been reported in field situations in some countries [14,15,16,17,18,19,20]. Alternative fumigants have been investigated, including sulfuryl fluoride [21], ethyl formate [22], hydrogen cyanide [23], and nitric oxide [24], but these have several disadvantages [22,23]. Thus, there is an increasing desire to use more toxicologically and environmentally benign chemicals in insect pest management. The use of naturally occurring toxins, such as some monoterpenes, rather than conventional synthetic pesticides is becoming more desirable in pest management programs because these compounds generally show rapid environmental biodegradation and lower toxicity to nontarget organisms, e.g., natural enemies, humans, and other vertebrates [25]. Monoterpenes, which are components of essential oils (EOs) found in many aromatic plants, are considered suitable compounds with which to develop new insecticides because they are typically safe, effective, and fully biodegradable [26]. Moreover, the toxicities of monoterpenes have been demonstrated against *P. interpunctella* as well as several other stored-product pests including *Sitophilus oryzae* (L.) (Coleoptera: Curculionidae), *Tribolium castaneum* (Herbst) (Coleoptera: Tenebrionidae), *Rhyzopertha dominica* (F.) (Coleoptera: Bostrichidae), and *Callosobruchus chinensis* (L.) (Coleoptera: Chrysomelidae) [27,28,29].

Methyl benzoate (MBe) is a floral volatile organic compound found within many plants including snapdragons and petunias [30]. It has a sweet, balsamic, spicy, and heady scent [31]; consequently, it has been used as a component of fragrances within the perfume industry [32]. In addition, MBe is known to biodegrade slowly in the atmosphere [33]. Recently, MBe has been shown to have contact toxicity against various insect pests including spotted wing drosophila, tobacco hornworms, brown marmorated stink bugs, diamondback moths, red imported fire ants, whiteflies, aphids, and mites [34,35,36,37,38]. Furthermore, the potential fumigation toxicity of MBe has been demonstrated on bed bugs and some stored-product insect pests [29,39,40,41]. However, its fumigation toxicity has yet to be evaluated in *P. interpunctella*.

This aim of this study was therefore to assess the effectiveness of MBe as a fumigant against adult *P. interpunctella* in the laboratory. Moreover, its efficacy relative to some commercially available monoterpenes, i.e., citronellal, linalool, 1,8 cineole, and limonene, was evaluated along with a positive control of ethyl formate.

## 2. Materials and Methods

### 2.1. Insects and Chemicals

The initial population of moths used in the experiments was acquired originally from home storage of grain products in Daegu, Korea, in 2019, and brought into the laboratory. The colony of *P. interpunctella* was maintained at the insect physiology laboratory, Kyungpook National University, Daegu, Korea, where they were reared at 27 °C ± 1 °C, 70% ± 5% relative humidity, with a 16:8 h light:dark photoperiod, on an artificial diet consisting of a mixture of wheat bran (410 gm), pollen (780 gm), honey (80 mL), glycerin (80 mL), water (10 mL), and methyl paraben (1.17 gm). During their maintenance, *P. interpunctella* colony was not exposed to any insecticides.

**Figure 1:**
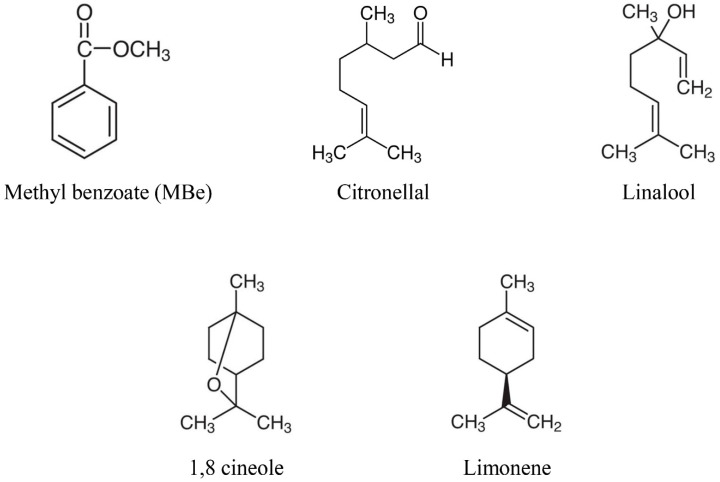
The chemical structures of methyl benzoate (MBe) and the other tested monoterpenes.

The commercially available compounds MBe (99%), citronellal (95%), linalool (95%), 1,8 cineole (99%), limonene (97%), and ethyl formate (97%) were purchased from Sigma-Aldrich (St. Louis, MO, USA) (Figure 1).

### 2.2. Fumigant Toxicity of MBe against Adults of P. interpunctella: Glass Bottle Bioassay

**Figure 2:**
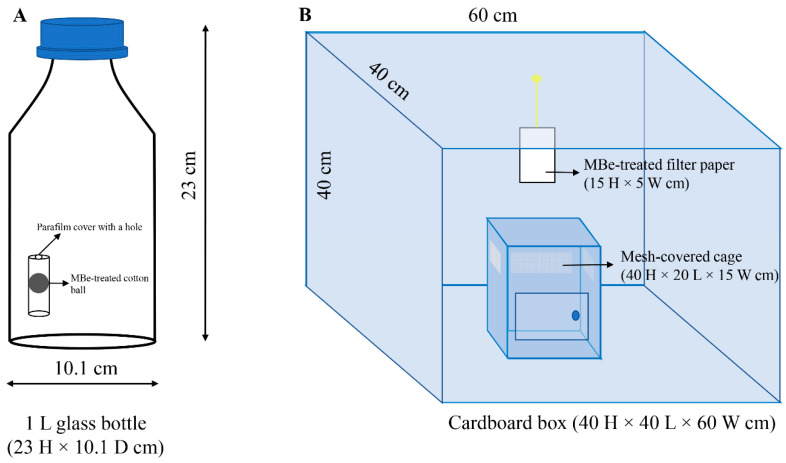
Experimental setups for testing the fumigation toxicity of methyl benzoate (MBe) against adults of *Plodia interpunctella.* (**A**) glass bottles (1 L) and (**B**) cardboard boxes were used as fumigation chambers.

Two different methods were employed to evaluate MBe fumigation toxicity on adults of *P. interpunctella*. First, MBe fumigation of *P. interpunctella* was conducted in 1-L glass bottles (Schott Duran), which were sealed and airtight to serve as fumigation chambers. Various MBe concentrations (0.1, 0.3, 0.5, and 1 μL/L air) were loaded onto small cotton balls without any solvent, whereas plain cotton balls served as blank controls; each of these was placed within a small (1.5 mL) Eppendorf plastic tube (Figure 2A). Different concentrations of ethyl formate (1, 3, 4, and 5 μL/L air) were applied in the same manner to serve as positive controls. Each Eppendorf plastic tube was sealed with Parafilm (Bemis Company Inc., Neenah, WI, USA), leaving only a small hole in the center to prevent direct contact with the tested insects and the treated cotton ball. The Eppendorf plastic tube containing the cotton balls were then placed to the bottom of the glass bottle. For experimentation, sets of 10 mixed-sex adults (<5 days old) were transferred from the stock culture to the glass bottle. All bottles were maintained in the growth chamber for 4 h at the temperature and RH conditions described in Section 2.1. The number of dead *P. interpunctella* moths in each bottle was counted at 1-h intervals during the 4-h exposure. Insects that were lying on their backs and/or unable to move upon prodding were considered dead. All treatments were replicated five times.

### 2.3. Fumigant Toxicity of MBe against Adults of P. interpunctella: Cardboard Box Bioassay

In a second bioassay, cardboard boxes (40 × 40 × 60 cm; volume ca. 96,000 cm^3^; Figure 2B) were used as fumigation chambers under the same laboratory conditions described in Section 2.1 to determine whether the effective MBe concentrations in the glass bottle assay retained their fumigant efficacy in a larger space. Filter paper (15 × 5 cm; Whatman No. 1, Maidstone, England) was first moistened uniformly by applying 1000 μL of MBe solution. Once dry, the filter paper was attached to the top of each cardboard box with adhesive tape. In each replicate, 20 mixed-sex *P. interpunctella* adults (<5 days old) were added to a mesh-covered insect proof cage (40 × 20 × 15 cm; Figure 2B), which was placed inside the cardboard box. Control boxes contained untreated filter paper. Each cardboard box was sealed with tape, and the mortality of the moths was assessed after 24 h. Each treatment was replicated five times.

### 2.4. Comparison of the Fumigation Toxicity of MBe with That of Other Monoterpenes

A series of bioassays was conducted as described in Section 2.2 and Section 2.3 to compare the fumigation toxicity of MBe to that of other monoterpenes (i.e., those considered “minimum-risk pesticides” [34]). To compare the fumigant toxicity of MBe, citronellal, linalool, 1,8 cineole, and limonene against adults of *P. interpunctella* in the glass bottle bioassay, we selected an effective concentration of 1 μL/L air. On the other hand, in the cardboard box bioassay, we compared only MBe and citronellal because the latter showed stronger fumigant toxicity than the other selected monoterpenes when used in the glass bottle bioassay. Each comparative bioassay was replicated five times, and mortality data were recorded as described in Section 2.2 and Section 2.3.

### 2.5. Statistical Analysis

All statistical analyses were performed in SAS 9.4 [42], while SigmaPlot 12.5 was used to construct all graphs. Two-way ANOVA followed by Dunnett’s post hoc test determined the interactions of MBe concentrations and exposure times, and variations between treatment groups were examined by one-way ANOVA followed by Tukey’s post hoc study (*p* < 0.05). Additionally, 95% confidence intervals were calculated. Abbott’s formula was not applied to bioassay data because control mortalities never exceeded 10%. To determine median lethal concentrations (LC_50_s) for *P. interpunctella* after 4 h as well as median lethal time (LT_50_s), log-probit analysis of data was applied.

## 3. Results

### 3.1. Fumigant Toxicity of MBe against Adults of P. interpunctella: Glass Bottle Bioassay

**Figure 3:**
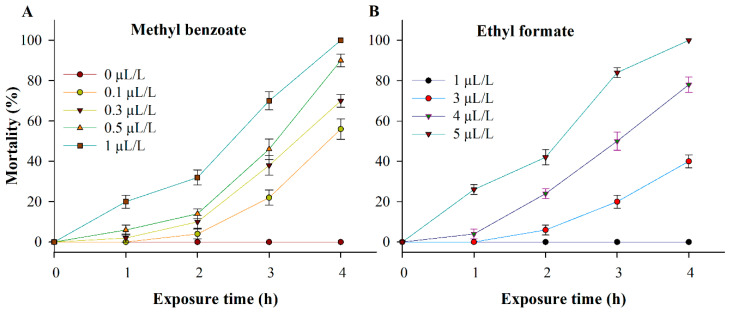
Fumigation toxicity of methyl benzoate (MBe) (**A**) and ethyl formate (positive control) (**B**) against adults of *Plodia interpunctella.* Adult mortality was evaluated at 1-h intervals during 4 h of exposure.

MBe fumigation was effective against *P. interpunctella* adults at all tested concentrations, with maximum mortality achieved by 1 μL MBe/L air following a 4-h exposure period (Figure 3A). The mortality of *P. interpunctella* adults was MBe concentration- and exposure time-dependent. After 4 h of exposure, maximum mortality was recorded at 56%, 70%, 90%, and 100% for MBe with 0.1, 0.3, 0.5, and 1 μL/L air, respectively (Figure 3A). For ethyl formate as the positive control, 100% mortality was observed with a concentration of 5 μL/L air at a 4-h exposure (Figure 3B).

The LT_50_ values for MBe at 0.1, 0.3, 0.5, and 1 μL/L air were 3.8 h (*χ*^2^ = 0.54, df = 2, *p* = 0.764), 3.3 h (*χ*^2^ = 7.31, df = 2, *p* = 0.026), 2.8 h (*χ*^2^ = 32.7, df = 2, *p* < 0.0001), and 2.0 h (*χ*^2^ = 34.8, df = 2, *p* < 0.0001), respectively (Table 1).

Probit analysis revealed that the LC_50_ value for MBe as a fumigant treatment against adults of *P. interpunctella* was 0.1 μL/L air (Table 2). Based on this LC_50_ value, MBe showed greater toxicity than the positive control ethyl formate (3.2 μL/L air).

As shown in Table 3, there were significant differences among the mortality effects of MBe concentrations (*F* = 184.57; df = 4, 80; *p* < 0.0001) and exposure times (*F* = 366.42; df = 3, 80; *p* < 0.0001). In addition, the interaction between MBe concentrations and exposure times also had a significant effect on the mortality of *P. interpunctella* (*F* = 26.405; df = 12, 80; *p* < 0.0001) (Table 3).

### 3.2. Fumigant Toxicity of MBe against Adults of P. interpunctella: Cardboard Box Bioassay

**Figure 4:**
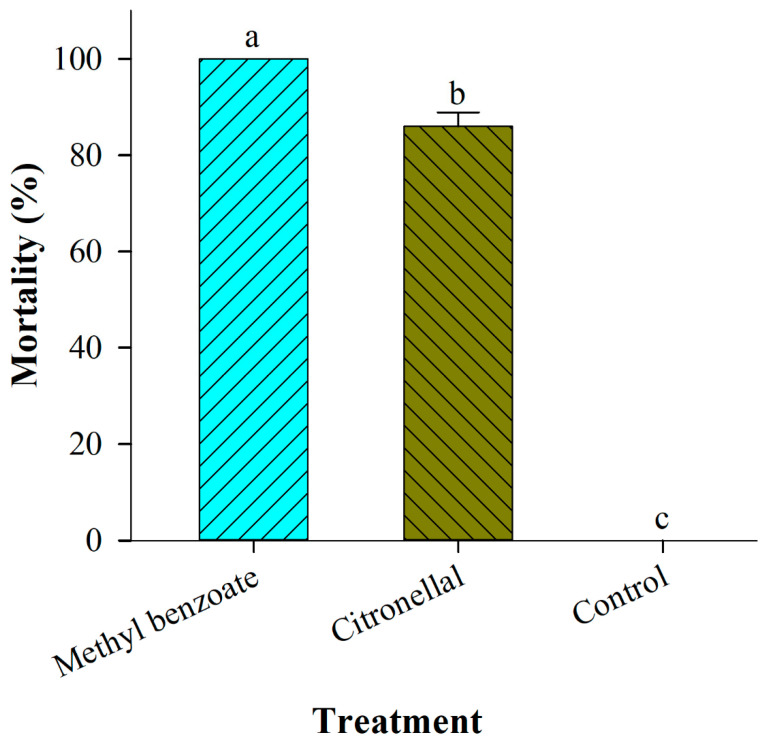
Mortality (%) of *Plodia interpunctella* adults contained within a large cardboard box (volume 96,000 cm^3^) after a 24-h fumigation exposure to 0.01 μL/cm^3^ (i.e., 1 mL) of methyl benzoate (MBe) or citronellal. The control boxes contained untreated filter paper. Vertical bars represent mean percentage ± SE. Different lowercase letters above bars represent significant differences at *p* < 0.05.

In the second (confirmatory) fumigation experiment, MBe showed stronger fumigation toxicity than citronella against adults of *P. interpunctella*. After 24 h of exposure to MBe at a concentration of 0.01 μL/cm^3^, 100% mortality was observed (Figure 4). In contrast, for the same concentration and exposure time, citronellal produced 86% mortality. There was a significant difference between the two tested compounds (*F* = 1034.82; df = 2, 12; *p* < 0.0001) (Figure 4).

### 3.3. Fumigant Toxicity of MBe Compared to That of Other Monoterpenes

**Figure 5:**
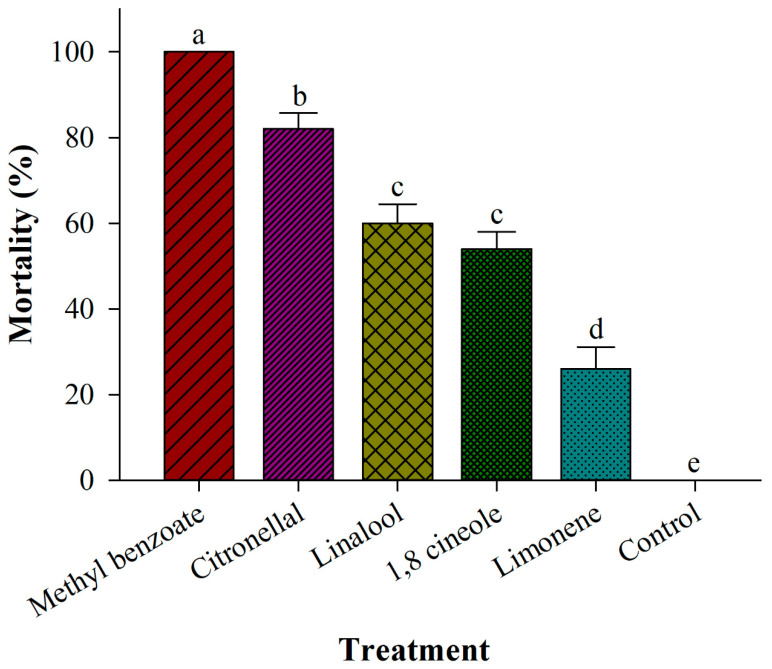
Effects of tested monoterpenes on the mortality of *Plodia interpunctella* adults after 4 h of exposure. Different lowercase letters above bars represent significant differences at *p* < 0.05.

Among all of the tested compounds, MBe had the highest fumigant toxicity against adults of *P. interpunctella*. After 4 h of exposure, MBe at a concentration of 1 μL/L air produced 100% mortality, whereas equivalent concentrations of citronellal, linalool, 1,8 cineole, and limonene produced 82%, 60%, 54%, and 26% mortality rates, respectively (Figure 5). There were significant differences among the effects of MBe and those of other monoterpenes (*F* = 104.77; df = 5, 24; *p* < 0.0001) (Figure 5).

## 4. Discussion

Naturally occurring essential oils are good candidates as safe alternatives to conventional fumigants because of their low mammalian and environmental toxicity, as well as their high volatility [39,41,43,44]. The present study demonstrated that MBe has potent fumigation toxicity against adults of *P. interpunctella*. Based on our laboratory toxicity data, MBe was more toxic than some commercially available monoterpenes, i.e., citronellal, linalool, 1,8 cineole, and limonene. In addition, our results indicated that the fumigation toxicity of MBe is concentration- and exposure time-dependent.

Morrison et al. [39] found that a 24-h exposure to high concentrations of MBe caused high fumigant toxicity against *R. dominica* and *T. castaneum*, whereas *Sitophilus zeamais* (Motschulsky) (Coleoptera: Cucurlionidae) and *Trogoderma variabile* (Ballion) (Coleoptera: Dermestidae) were less susceptible. Park et al. [29] also found that MBe was highly toxic to *C. chinensis* at 5.36 mg/L after 24 h of exposure. In the current study, MBe showed potent fumigant toxicity against adults of *P. interpunctella* with LT_50_ values as low as 2 h with 1 μL/L air. Similarly, Yang et al. [41] reported that the LT_50_ value of MBe on *S. oryzae* was 6.21 h, while the LT_50_ values against *Frankliniella occidentalis* (Pergande) (Thysanoptera: Thripidae) and *Rhizoglyphus* spp. (Sarcoptiformes: Acaridae) were 0.49 and 19.07 h, respectively [41]. The differences between our results and those of other studies are likely due to differences in the insects tested.

Monoterpenes demonstrate strong fumigant action against pest species due to their high volatility [12,45,46]. Among the monoterpenes used against the adults of *P. interpunctella* in the present study, citronellal had the highest fumigation activity; however, MBe showed stronger fumigation toxicity than citronellal in both of our laboratory bioassays. The toxic effect of a substance was not solely dependent on its volatility but also depended on toxicokinetic steps and unique target-receptor interactions [46,47,48] as well as physiochemical properties (Table 4) [49].

In previous studies, the fumigant toxicities of the monoterpenes tested here were assessed against other stored-product pests [45,51,52]. For instance, limonene, and linalool had the highest fumigation toxicities against adults of *S. oryzae* and *T. castaneum* with LC_50_ values of 26.92, 52.78, and 33.37, >100 mg/L of air, respectively [45]. Limonene also showed fumigation toxicities against adults of *R. dominica*, *S. oryzae*, and *T. castaneum* in another study, with LD_95_ values of 7.04, 12.75, and 11.11 mg/L air, respectively [52]. Besides, 1,8 cineole was reported to produce >90% mortality to *S. oryzae*, *T. castaneum*, *R. dominica,* and *Tenebrio molitor* (L.) (Coleoptera: Tenebrionidae) with LD_95_ values of 22.8, 15.3, 9.5, and 5.7 μL/L of air, respectively [27,53]. Both citronellal and limonene also caused 100% mortality of *Oryzaephilus surinamensis* (L.) (Coleoptera: Silvanidae) at 50 μg/mL air [51].

In the present study, the vapor pressure of MBe was 0.38 mmHg at 25 °C (Table 4), while the calculated saturated vapor concentration of the compound is theoretically 500 ppm [41]. Recently, Yang et al. [41] reported that the vapor concentrations of MBe in a headspace chamber at 25 °C for at 2 and 72 h were 438 and 435 ppm, respectively. Thus, MBe concentrations were stable at 2–72 h and close to 500 ppm (theoretically). Only a small amount of liquid MBe was vaporized by the end of the fumigation treatment, likely because of the low vapor pressure of MBe. In the current study, the low effective concentration of MBe also suggested its high efficacy against adults of *P. interpunctella*.

Compared to most other gas fumigants, MBe is much less volatile and may therefore have less capacity to penetrate products such as vegetables and stored goods. However, Yang et al. [41] reported that MBe is useful as a fumigant against insects on both fresh and stored products and showed that MBe fumigation could combat postharvest pests. Moreover, they also found that MBe fumigation had no adverse effect on the quality of apples in terms of color, weight, and firmness.

## 5. Conclusions

In conclusion, our findings indicate that MBe has high fumigant toxicity against adults of *P. interpunctella*. Our findings also illustrate that MBe stands out among other potential natural biofumigants as a strong candidate to protect stored products against insect pests (here *P. interpunctella*); thus, MBe may be a safer and perhaps more effective alternative to traditional fumigants. However, further research is required to assess and promote the natural evaporation of MBe in commercial-scale trials, improve control efficiency, and develop protocols for commercial-scale treatment.

## Figures and Tables

**Table 1:** Probit analysis of time-mortality responses of *Plodia interpunctella* adults exposed to various concentrations of methyl benzoate (MBe).

MBe Concentrations (μL/L air)	LT_50_ (h)	95% CI (Lower–Upper)	Slope (SEM)	*X*^2^ (df)
0.1	3.8	(3.6–4.2)	6.5 (0.85)	0.54 (2)
0.3	3.3	(2.3–16.4)	4.9 (1.05)	7.31 (2)
0.5	2.8	(1.33–6.0)	4.9 (1.85)	32.7 (2)
1.0	2.0	-	3.9 (1.47)	34.8 (2)

CI = confidence interval; df = degrees of freedom.

**Table 2:** Probit analysis of concentration–mortality responses of *Plodia interpunctella* adults exposed to various concentrations of methyl benzoate (MBe) and ethyl formate.

Treatment	LC_50_ (μL/L air)	95% CI (Lower–Upper)	Slope (SEM)	*X*^2^ (df)
Methyl benzoate	0.1	-	1.8 (0.23)	12.16 (2)
Ethyl formate	3.2	(2.1–3.7)	10.4 (1.87)	4.98 (2)

CI = confidence interval; df = degrees of freedom; LC_50_ values were calculated by using the 4-h mortality data.

**Table 3:** Two-factor repeated measures ANOVA results testing the effects of different concentrations and exposure times on the fumigation toxicity of methyl benzoate (MBe) against adults of *Plodia interpunctella*.

Source	Type III Sum of Squares	df	Mean Square	F	*p*
Corrected Model	100,180	19	5272.632	113.39	<0.0001
Intercept	84,100	1	84,100	1808.6	<0.0001
MBe concentrations	34,330	4	8582.5	184.57	<0.0001
Exposure times	51,116	3	17,038.667	366.423	<0.001
MBe concentrations × Exposure times	14,734	12	1227.833	26.405	<0.001
Error	3720	80	46.5		
Total	188,000	100			
Corrected Total	103,900	99			

df = degrees of freedom.

**Table 4:** Physiochemical properties of the tested compounds.

Compound	Vapor Pressure (mmHg at 25 °C)	Boiling Point (°C at 760 mmHg)
Methyl benzoate	0.38	199.0
Citronellal	0.28	207.0
Linalool	0.10	198.0
1,8 cineole	1.60	176.0
Limonene	1.50	175.0

Data were obtained from Phillips et al. [50] and the PubChem Open Chemistry Database (https://pubchem.ncbi.nlm.nih.gov/).

## Data Availability

There is no supplementary information to reveal, all the information is contained in this manuscript.

## References

[1] Rees D.P. (2007). Insects of Stored Grain: A Pocket Reference.

[2] Nasir M.F., Ulrichs C., Prozell S., Scholler M. (2017). Laboratory studies on parasitism of *Plodia interpunctella* (Hübner) (Lepidoptera: Pyralidae) by two species of *Trichogramma* Westwood (Hymenoptera: Trichogrammatidae) in different grains, and evaluation of traps for their monitoring. J. Stored Prod. Res..

[3] Loschiavo S.R., Okumura G.T. (1979). A survey of stored product insects in Hawaii. Proc. Hawaii. Entomol. Soc..

[4] Arbogast R.T., Kendra P.E., Mankin R.W., McGovern J.E. (2000). Monitoring insect pests in retail stores by trapping and spatial Analysis. J. Econ. Entomol..

[5] Roesli R., Subramanyam B., Campbell J.F., Kemp K. (2003). Stored-product insects associated with a retail pet store chain in Kansas. J. Econ. Entomol..

[6] Hagstrum D.W., Klejdysz T., Subramanyam B., Nawrot J. (2013). Atlas of Stored-Product Insects and Mites.

[7] Mohandass S., Arthur F.H., Zhu K.Y., Throne J.E. (2007). Biology and management of *Plodia interpunctella* (Lepidoptera: Pyralidae) in stored products. J. Stored Prod. Res..

[8] Hubert J., Stejskal V., Athanassiou C.G., Throne J.E. (2018). Health hazards associated with arthropod infestation of stored products. Annu. Rev. Entomol..

[9] Mbata G.N., Shapiro-Ilan D.I. (2010). Compatibility of *Heterorhabditis indica* (Rhabditida: Heterorhabditidae) and *Habrobracon hebetor* (Hymenoptera: Braconidae) for biological control of *Plodia interpunctella* (Lepidoptera: Pyralidae). Biol. Control.

[10] Kim H., Yu Y.S., Lee K.Y. (2014). Synergistic effects of heat and diatomaceous earth treatment for the control of *Plodia interpunctella* (Lepidoptera: Pyralidae). Entomol. Res..

[11] Hagstrum D.W., Phillips T.W. (2017). Evolution of stored-product entomology: Protecting the world food supply. Annu. Rev. Entomol..

[12] Rajendran S., Sriranjini V. (2008). Plant products as fumigants for stored-product insect control. J. Stored Prod. Res..

[13] Aulicky R., Stejskal V., Frydova B., Athanassiou C.G. (2015). Susceptibility of two strains of the confused flour beetle (Coleoptera: Tenebrionidae) following phosphine structural mill fumigation: Effects of concentration, temperature, and flour deposits. J. Econ. Entomol..

[14] Taylor R.W.D. (1989). Phosphine-a major fumigant at risk. Int. Pest Control.

[15] Sayaboc P., Gibe A., Caliboso F. (1998). Resistance of *Rhizopertha dominica* (F.) (Coleoptera: Bostrychidae) to phosphine in the Philippines. Philipp. Entomol..

[16] Benhalima H., Chaudhry M.Q., Mills K.A., Price N.R. (2004). Phosphine resistance in stored-product insects collected from various grain storage facilities in Morocco. J. Stored Prod. Res..

[17] Pimentel M.A.G., Faroni L.R.D., Guedes R.N.C., Sousa A.H., Tótola M.R. (2009). Phosphine resistance in Brazilian populations of *Sitophilus zeamais* Motschulsky (Coleoptera: Curculionidae). J. Stored Prod. Res..

[18] Jagadeesan R., Collins P.J., Daglish G.J., Ebert P.R., Schlipalius D.I. (2012). Phosphine resistance in the rust red flour beetle, *Tribolium castaneum* (Coleoptera: Tenebrionidae): Inheritance, gene interactions and fitness costs. PLoS ONE.

[19] Opit G.P., Phillips T.W., Aikins M.J., Hasan M.M. (2012). Phosphine resistance in *Tribolium castaneum* and *Rhyzopertha dominica* from stored wheat in Oklahoma. J. Econ. Entomol..

[20] Afful E., Elliott B., Nayak M.K., Phillips T.W. (2018). Phosphine resistance in North American field populations of the lesser grain borer, *Rhyzopertha dominica* (Coleoptera: Bostrichidae). J. Econ. Entomol..

[21] Barakat D., Flingelli G., Reichmuth C. (2011). Lethal effect of sulfuryl fluoride on eggs of different age of the Indian meal moth *Plodia interpunctella* (Hübner)—Demonstration of the no constancy of the ct products for control. J. Fur Kult..

[22] Haritos V.S., Damcevski K.A., Dojchinov G. (2006). Improved efficacy of ethyl formate against stored grain insects by combination with carbon dioxide in a ‘dynamic’ application. Pest Manag. Sci..

[23] Rambeau M., Benitez D., Dupuis S., Ducom P., Donahaye E.J., Navarro S., Leesch J.G. (2001). Hydrogen cyanide as an immediate alterative to methyl bromide for structural fumigations. Proceedings of the International Conference on Controlled Atmosphere and Fumigation in Stored Products.

[24] Liu Y.B. (2013). Nitric oxide as a potent fumigant for postharvest pest control. J. Econ. Entomol..

[25] Copping L.G., Duke S.O. (2007). Natural products that have been used commercially as crop protection agents. Pest Manag. Sci..

[26] Xie Y., Wang K., Huang Q., Lei C. (2014). Evaluation toxicity of monoterpenes to subterranean termite, *Reticulitermes chinensis* Snyder. Ind. Crop. Prod..

[27] Lee B.-H., Annis P.C., Tumaalii F., Choi W.-S. (2004). Fumigant toxicity of essential oils from the Myrtaceae family and 1,8-cineole against 3 major stored-grain insects. J. Stored Prod. Res..

[28] Borzoui E., Naseri B., Abedi Z., Karimi-Pormehr M.S. (2016). Lethal and sublethal effects of essential oils from *Artemisia khorassanica* and *Vitex pseudo-negundo* against *Plodia interpunctella* (Lepidoptera: Pyralidae). Environ. Entomol..

[29] Park C.G., Shin E., Kim J. (2016). Insecticidal activities of essential oils, *Gaultheria fragrantissima* and *Illicium verum*, their components and analogs against *Callosobruchus chinensis* adults. J. Asia-Pac. Entomol..

[30] Negre F., Kish C.M., Boatright J., Underwood B., Shibuya K., Wagner C., Clark D.G., Dudareva N. (2003). Regulation of methyl benzoate emission after pollination in snapdragon and petunia flowers. Plant Cell.

[31] Choudhary M.I., Naheed N., Abbaskhan A., Musharraf S.G., Siddiqui H. (2008). Atta-Ur-Rahman Phenolic and other constituents of fresh water fern Salvinia molesta. Phytochemistry.

[32] Bickers D.R., Calow P., Greim H.A., Hanifin J.M., Rogers A.E., Saurat J.-H., Glenn Sipes I., Smith R.L., Tagami H. (2003). The safety assessment of fragrance materials. Regul. Toxicol. Pharmacol..

[33] Atkinson R. (1987). A structure-activity relationship for the estimation of rate constants for the gas-phase reactions of OH radicals with organic compounds. Int. J. Chem. Kinet..

[34] Feng Y., Zhang A. (2017). A floral fragrance methyl benzoate is an efficient green pesticide. Sci. Rep..

[35] Mostafiz M.M., Jhan P.K., Shim J.K., Lee K.Y. (2018). Methyl benzoate exhibits insecticidal and repellent activities against *Bemisia tabaci* (Gennadius) (Hemiptera: Aleyrodidae). PLoS ONE.

[36] Mostafiz M.M., Hassan E., Shim J.K., Lee K.Y. (2019). Insecticidal efficacy of three benzoate derivatives against *Aphis gossypii* and its predator *Chrysoperla carnea*. Ecotoxicol. Environ. Saf..

[37] Mostafiz M.M., Shim J.K., Hwang H.S., Bunch H., Lee K.Y. (2020). Acaricidal effects of methyl benzoate against *Tetranychus urticae* Koch (Acari: Tetranychidae) on common crop plants. Pest Manag. Sci..

[38] Chen J., Rashid T., Feng G., Feng Y., Zhang A., Grodowitz M.J. (2019). Insecticidal activity of methyl benzoate analogs against red imported fire ants, *Solenopsis invicta* (Hymenoptera: Formicidae). J. Econ. Entomol..

[39] Morrison W.R., Larson N.L., Brabec D., Zhang A. (2019). Methyl benzoate as a putative alternative, environmentally friendly fumigant for the control of stored product insects. J. Econ. Entomol..

[40] Larson N.R., Zhang A., Feldlaufer M.F. (2020). Fumigation activities of ethyl benzoate and its derivatives against the common bed bug (Hemiptera: Cimicidae). J. Med. Entomol..

[41] Yang X., Liu Y.-B., Feng Y., Zhang A. (2020). Methyl benzoate fumigation for control of post-harvest pests and its effects on apple quality. J. Appl. Entomol..

[42] SAS Institute Inc. (2016). Base SAS 9.4 Procedures Guide: High-Performance Procedures.

[43] Shaaya E., Kostjukovski M., Eilberg J., Sukprakarn C. (1997). Plant oils as fumigants and contact insecticides for the control of stored-product insects. J. Stored Prod. Res..

[44] Kedia A., Prakash B., Mishra P.K., Singh P., Dubey N.K. (2015). Botanicals as eco friendly biorational alternatives of synthetic pesticides against *Callosobruchus* spp. (Coleoptera: Bruchidae)—A review. J. Food Sci. Technol..

[45] Abdelgaleil S.A.M., Mohamed M.I.E., Badawy M.E.I., El-arami S.A.A. (2009). Fumigant and contact toxicities of monoterpenes to *Sitophilus oryzae* (L.) and *Tribolium castaneum* (Herbst) and their inhibitory effects on acetylcholinesterase activity. J. Chem. Ecol..

[46] Zhang Z., Yang T., Zhang Y., Wang L., Xie Y. (2016). Fumigant toxicity of monoterpenes against fruitfly, *Drosophila melanogaster*. Ind. Crop. Prod..

[47] Toloza A.C., Zygadlo J., Cueto G.M., Biurrun F., Zerba E., Picollo M.I. (2006). Fumigant and repellent properties of essential oils and component compounds against permethrin-resistant *Pediculus humanus* capitis (Anoplura: Pediculidae) from Argentina. J. Med. Entomol..

[48] Gaire S., Scharf M.E., Gondhalekar A.D. (2019). Toxicity and neurophysiological impacts of plant essential oil components on bed bugs (Cimicidae: Hemiptera). Sci. Rep..

[49] Jesser E.N., Werdin-González J.O., Murray A.P., Ferrero A.A. (2017). Efficacy of essential oils to control the Indian meal moth, *Plodia interpunctella* (Hübner) (Lepidoptera: Pyralidae). J. Asia-Pac. Entomol..

[50] Phillips A.K., Appel A.G., Sims S.R. (2010). Topical toxicity of essential oils to the German cockroach (Dictyoptera: Blattellidae). J. Econ. Entomol..

[51] Lee S., Peterson C.J., Coats J.R. (2003). Fumigation toxicity of monoterpenoids to several stored product insects. J. Stored Prod. Res..

[52] Tripathi A.K., Prajapati V., Khanuja S.P.S., Kumar S. (2003). Effect of d-limonene on three stored-product beetles. J. Econ. Entomol..

[53] Lima R.K., Cardoso M.D., Moraes J.C., Carvalho S.M., Rodrigues V.G., Guimarães L.G.L. (2011). Chemical composition and fumigant effect of essential oil of *Lippia sidoides* Cham. and monoterpenes against *Tenebrio molitor* (L.) (Coleoptera: Tenebrionidae). Ciência E Agrotecnol..

